# Supplementary material for: SECAPR—a bioinformatics pipeline for the rapid and user-friendly processing of targeted enriched Illumina sequences, from raw reads to alignments
Source: PeerJ. 2018 Jul 13;6:e5175. doi: 10.7717/peerj.5175 (PMC6047508; doi:10.7717/peerj.5175)
Supplement: Appendix S1 — Detailed description of the lab workflow. [file peerj-06-5175-s001.pdf]

Library Preparation and Sequencing

Total genomic DNA was extracted from dry leaf samples using NucleoSpin® Plant II (Machery-Nagle) and following the manufacturer's protocols. Genomic DNA was sonicated with a Covaris S220 instrument (Covaris, Woburn, Massachusetts, USA). DNA libraries were constructed with the NEXTflexTM DNA Rapid Sequencing Kit and NEXTflexTM DNA Barcodes (BIOO Scientific, Austin, Texas, U.S.A.) following the manufacturer's protocol. Agencourt AMPure XP magnetic beads (Beckman Coulter) were used for fragment size selection to exclude fragments under 400 bp and over 600 bp. For better yield, a PCR run of 14 cycles was performed on the libraries, using the reagents provided with the kits, with the following program: 98°C, 2'; 14x(98°C, 30"; 65°C, 30"; 72°C, 60"); 72°C, 4'. Purification steps recommended by the manufacturer were done with the QIAquick Purification kit (QIAGEN, Hilden, Germany). Targeted loci were captured using the palm probes designed by Heyduk et al (2015) to target 176 genes divided into 837 exons. DNA concentration and fragment size were measured at different stages using the Qubit 3.0 Fluorometer (Thermo Fisher Scientific, Waltham, Massachusetts, U.S.A.) and TapeStation (Agilent Technologies, Santa Clara, California, U.S.A.) respectively. Captured libraries were sequenced by the Swedish Science for Life Laboratory (SciLifeLab), on a MiSeq v3 platform from Illumina (San Diego, California, USA), producing 300 bp paired-end reads.
