# Supplementary material for: SECAPR—a bioinformatics pipeline for the rapid and user-friendly processing of targeted enriched Illumina sequences, from raw reads to alignments
Source: PeerJ. 2018 Jul 13;6:e5175. doi: 10.7717/peerj.5175 (PMC6047508; doi:10.7717/peerj.5175)
Supplement: Appendix S2 [file peerj-06-5175-s002.pdf]

Phylogenetic Inference under the Multispecies Coalescent (MSC) model

Using the SECAPR *locus_selection* function we selected the 50 exon loci with the highest read coverage across all samples after reference-based assembly. We then applied the SECAPR *phase_alleles* function in order to generate two allele sequences for each sample and locus. The resulting phased allele sequences of all samples were aligned for each locus, resulting into 50 allele Multiple Sequence Alignments (MSAs). These 50 MSAs were analyzed using the STACEY (Graham Jones 2017; G. Jones et al. 2015) plugin in BEAST2 (v.2.4.4) (Bouckaert et al. 2014). Using the BEAUTI GUI (v.2.4.4), we unlinked the site models, clock models and gene trees of all loci. We chose a JC substitution model for all loci, a fixed strict clock rate of 1 for one random locus and estimated all other clock rates relative to this locus (strict clock). We chose the STACEY-specific BirthDeathCollapse species tree model with a collapse height of 1e-5. This tree-model allows taxon-assignment free analyses under the MSC model, which allows to observe every sequence as an individual tip in the species tree (rather than having to assign sequences to expected clusters (= species) prior to analysis. Further priors were: bdcGrowthRate = log normal(M=4.6, S=1.5); collapseWeight = beta (alpha=2, beta=2); popPriorScale = log normal(M=-7, S=2); relativeDeathRate = beta (alpha=1.0, beta=1.0).

We set the MCMC for 1 billion generations, logging every 100,000 generations. After approximately 500 million generations all parameters had reached convergence (assessed with Tracer v.1.6, Rambaut et al. 2013) and the MCMC was stopped (approximately 80 hours on a Mac Pro, Late 2013, 3.5 GHz 6-Core Intel Xeon E5 processor). The resulting distribution of species trees was summarized with TreeAnnotator (v2.4.4), using mean heights and excluding 10% burn-in. We then used the SpeciesDelimitationAnalyser (part of the STACEY distribution) to calculate the posterior probability for each pair of species of belonging to the same cluster. This probability was calculated from the complete posterior tree distribution (excl. 10% burn-in), using a user-defined collapse-height value of 1e-5.

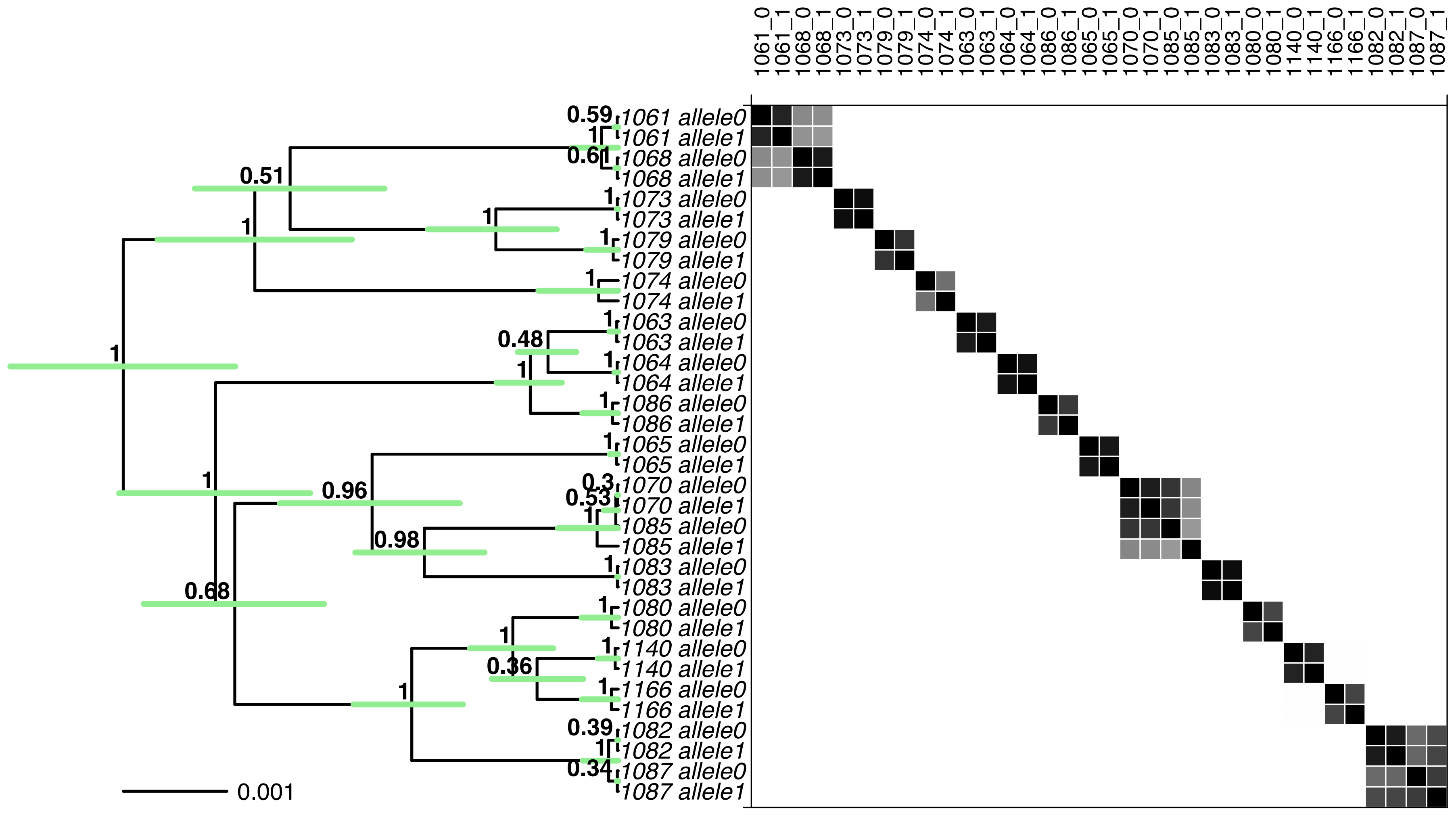

1061 allele0
1061 allele1
1068 allele0
1068 allele1
1073 allele0
1073 allele1
1079 allele0
1079 allele1
1074 allele0
1074 allele1
1063 allele0
1063 allele1
1064 allele0
1064 allele1
1086 allele0
1086 allele1
1065 allele0
1065 allele1
1070 allele0
1070 allele1
1085 allele0
1085 allele1
1083 allele0
1083 allele1
1080 allele0
1080 allele1
1140 allele0
1140 allele1
1166 allele0
1166 allele1
1082 allele0
1082 allele1
1087 allele0
1087 allele1
0.59
0.61
0.51
0.48
0.96
0.3
0.53
0.98
0.68
0.36
0.39
0.34
1
0.001
1061_0
1061_1
1068_0
1068_1
1073_0
1073_1
1079_0
1079_1
1074_0
1074_1
1063_0
1063_1
1064_0
1064_1
1086_0
1086_1
1065_0
1065_1
1070_0
1070_1
1085_0
1085_1
1083_0
1083_1
1080_0
1080_1
1140_0
1140_1
1166_0
1166_1
1082_0
1082_1
1087_0
1087_1

References

Bouckaert, Remco, Joseph Heled, Denise Kühnert, Tim Vaughan, Chieh-Hsi Wu, Dong Xie, Marc A. Suchard, et al. 2014. "BEAST 2: A Software Platform for Bayesian Evolutionary Analysis." Edited by Andreas Prlic. *PLoS Computational Biology* 10 (4). Public Library of Science: e1003537. doi:10.1371/journal.pcbi.1003537.

Jones, G., Z. Aydin, B. Oxelman, Aydin Z., Baele G., Belfiore N.M., de Queiroz K., et al. 2015. "DISSECT: An Assignment-Free Bayesian Discovery Method for Species Delimitation under the Multispecies Coalescent." *Bioinformatics* 31 (7). Oxford University Press: 991–98. doi:10.1093/bioinformatics/btu770.

Jones, Graham. 2017. "Algorithmic Improvements to Species Delimitation and Phylogeny Estimation under the Multispecies Coalescent." *Journal of Mathematical Biology* 74 (1–2): 447–67. doi:10.1007/s00285-016-1034-0.

Rambaut, A, Marc A. Suchard, Walter Xie, and AJ Drummond. 2013. "Tracer v1.6."
